# Foreign Correspondence

**Published:** 1889-08

**Authors:** 


### Foreign Correspondence.

To the Editor:

Your kind letter just to hand. I, like you, have been overworking somewhat, so much so that I have had to "knock off," and therefore it was not with an unmixed pleasure that I read your invitation to become a foreign correspondent of the International Dental Journal. There are some requests one cannot refuse, and this is one of them. Because, first, I appreciate the compliment, and I trust the distinction of being associated with so important a dental journal will be advantageous; second, I believe that from my position on the Representative Board of the British Dental Association, and from my being thoroughly in touch and communication with the representative men in our profession, I can be of some service to both countries.

The position of the American dentist in England is at present extremely unpleasant, on account of the unprofessional conduct of some holders of American diplomas,—real or assumed. We are making an organized effort by way of protest, of which more anon.

I enclose a specimen of the style of thing that makes "American dentistry" stink in the nostrils of so many good men here.

THE FOLLOWING ARE THE ADDRESSES OF THE AMERICAN DENTAL INSTITUTE (Limited).
55, ST. JAMES'-STREET, S.W. (near Piccadilly).
34, THURLOE-SQUARE, S.W.
44, FINSBURY-SQUARE, E.C.
BRIGHTON, 123, KING'S-ROAD, and
BOURNEMOUTH, Glen Fern Tower, Old Christchurch road.
Attendance, Nine till Six Daily.
Pamphlet free on application to the Secretary, at each address.

AMERICAN TOOTH CROWN COMPANY
(Reg.), 128, New Bond-street, W.
Established for the practice of GENUINE AMERICAN DENTISTRY, at uniform and moderate fees, by AMERICAN DENTISTS, GRADUATES of RECOGNIZED AMERICAN COLLEGES. Specialties—The Adjustment of Artificial Teeth Without any Plates or Wires whatsoever. All teeth, however badly decayed, permanently saved. Loose teeth tightened, and painless treatment. The privacy of the most refined dental practice is observed throughout the establishment. No appointment or fee necessary for consultation.
Hours Nine to Six.
Pamphlet free on application, 128, New Bond-street, W. (two doors from Grosvenor-street).

CROWN BRIDGE and BAR, by Dr. G. H. JONES. Like many other branches of study bearing on the health and comfort of humanity, the Dental Art has undergone great improvements, and in his pamphlet Dr. G. H. JONES shows that instead of its being delusive to speak of Painless Dentistry, it is as much an accomplished fact as the swift locomotive, the electric light, or the telephone.

The Crown System is the placing an artificial Crown of Gold or Porcelain upon the remaining portion of a tooth, thereby restoring its original contour and preventing further decay or pain. The bridge and bar facilitates the adjustment of teeth to the various conditions of the mouth without inconvenience.

Her Majesty's Surgeon-Dentist writes: "Dr. G. H. Jones.—My mastication is now perfect and articulation quite distinct. Your teeth are the best, safest, and most life-like, and your system is the perfection of painless dentistry. In recognition of your valuable services rendered to me, you are at liberty to use my name.—(Signed) S. G. HUTCHINS."

Dr. G H. JONES, who is a Graduate of the oldest Dental College in the United States, has an American as well as a European and Colonial reputation for successful Dentistry, which is confirmed by a certificate from the most eminent dentist in America, Dr. William H. Atkinson, M.D., D.D.S., of New York City, known as "The Father of American Dentistry," who writes: "Upon proper investigation as to the knowledge and ability of Dr. G. H. Jones as a Dentist in the presence of practical cases in my chair and otherwise, I am satisfied that he is qualified far above the mass of successful practitioners as I know them in America and Europe." The modern improvements render the fitting of teeth a painless and easy operation, which is fully explained in the new pamphlet "Painless and Perfect Dentistry," by Dr. G. H. JONES, F.R.S., Surgeon-Dentist and Doctor of Dental Surgery, 57, Great Russell-street, London, who will forward it by post to any address. Pamphlet and consultation free.

The Hutchins testimonial is said to be genuine, being the production of an old dentist of some repute, who is said to have held office under one of the lord lieutenants of Ireland, but who had all gone to the bad through drink. Her Majesty's surgeon-dentist is Sir Edwin Saunders, and, by appointment, Dr. John Smith, LL.D., in Scotland.

As to the other, can it be genuine? I should like to know.[1]

Recorrespondence,—let me have an idea as to when our mails reach you, and the convenient as well as your *latest* day for going to press.

Wishing you all success in your new and arduous undertaking,

I am, very faithfully yours,

GEORGE CUNNINGHAM.

MERLIN HALL, CAMBRIDGE, ENG.

---

[1] Dr. Atkinson denies all knowledge whatsoever of Dr. Jones, and says that the testimonial is a base fabrication.—ED.

